# What Remote PPG Oximetry Tells Us about Pulsatile Volume?

**DOI:** 10.3390/biomedicines12081784

**Published:** 2024-08-06

**Authors:** Gennadi Saiko

**Affiliations:** Department of Physics, Toronto Metropolitan University, Toronto, ON M5B 2K3, Canada; gsaiko@torontomu.ca

**Keywords:** photoplethysmography, microcirculation, pulse oximetry, perfusion

## Abstract

While pulse oximetry using remote photoplethysmography (rPPG) is used in medicine and consumer health, sound theoretical foundations for this methodology are not established. Similarly to traditional pulse oximetry, rPPG oximetry uses two wavelengths to calculate the tissue oxygenation using the so-called ratio-of-ratios, *R*. However, the relationship between *R* and tissue oxygenation has not been derived analytically. As such, rPPG oximetry relies mostly on empirical methods. This article aimed to build theoretical foundations for pulse oximetry in rPPG geometry. Using the perturbation approach in diffuse approximation for light propagation in tissues, we obtained an explicit expression of the AC/DC ratio for the rPPG signal. Based on this ratio, the explicit expression for “ratio-of-ratios” was obtained. We have simulated the dependence of “ratio-of-ratios” on arterial blood saturation across a wide range (SaO2 = 70–100%) for several commonly used R/IR light sources (660/780, 660/840, 660/880, and 660/940 nm) and found that the obtained relationship can be modeled by linear functions with an extremely good fit (*R*^2^ = 0.98–0.99) for all considered R/IR pairs. Moreover, the location of the pulsatile volume can be extracted from rPPG data. From experimental data, we found that the depth of blood pulsations in the human forehead can be estimated as 0.6 mm on the arterial side, which points to the papillary dermis/subpapillary vascular plexus origin of the pulsatile volume.

## 1. Introduction

While pulse oximetry is ubiquitous in medicine and consumer health, the strict foundations for this methodology are not well established. As such, it relies mostly on empirical methods, which is particularly true for remote photoplethysmography (rPPG).

The typical approach in pulse oximetry is to use two wavelengths and calculate the oxygenation using the so-called ratio-of-ratios, *R*.

(1) R=AC1DC1AC2DC2

Here, *AC* and *DC* refer to the amplitude of the AC component and the DC components of the measured signal; 1 and 2 refer to different wavelengths. Typically, the wavelengths are selected in red in the spectrum’s near-infrared (NIR) ranges, where oxyhemoglobin and deoxyhemoglobin have very different absorptions. For example, 660 nm and 940 nm are commonly used in commercial pulse oximeters [1]. 

The dependence of peripheral oxygen saturation (*S_p_O*2) on the “ratio-of-ratios” *R* in pulse oximetry is typically modeled using the linear relationship (see, for example, [1])

(2) $$SpO2=C_{1}-C_{2}R$$

Here, *C*_1_ and *C*_2_ are factors determined using a calibration procedure.

The same approach is used in rPPG pulse oximetry. For example, Humphrey et al. [2] observed pulse waveforms at 760 and 880 nm on 10 healthy volunteers and concluded that they could be used to extract SpO2 remotely. Shao et al. [3] found that the use of orange (611 nm) and NIR (880 nm) provides the best SNR for remote PPG pulse oximetry. Verkruysse et al. [4] showed that a single universal calibration curve with acceptable spread between individuals could be achieved by using 660 nm (red) and 840 nm (NIR) light. Much effort is dedicated to using only the visible spectrum range for remote pulse oximetry, as it allows for much simpler setups, like smartphone cameras. For example, Moco and Verkruysse [5], in addition to red (675 nm) and NIR (840 nm), used green (580 nm) as a potential substitution for NIR lights. They extracted the arterial blood oxygen saturation (SpO2) of 46 healthy adults. They found that SpO2 can be calibrated under controlled conditions with red and green light, but the accuracy is less than that of SpO2, as estimated in the usual red–NIR window.

However, we have identified a critical gap in the current knowledge. Namely, there is no sound analytical justification for an ability to extract tissue oxygenation in rPPG geometry.

In particular, while the use of “ratio-of-ratios” is typically explained using the Beer–Lambert model, oxygenation cannot be derived directly from physical and physiological considerations of light absorption in oxy- and deoxyhemoglobins, based on the Beer–Lambert law [6]. In particular, the ability to extract oxygenation is based on the primary assumption that the optical pathlengths for both wavelengths are identical. While it may be a reasonable assumption for traditional (transmissive mode) pulse oximeters [7], it is definitely not the case in remote PPG, where pathlengths linearly depend on the penetration depths [8], which are very different for red and infrared light [9,10]. Therefore, in practice, even transmissive mode commercial pulse oximeters use an empirical relationship, where the relationship between *R* and *S_a_O*2 is determined experimentally for each type of pulse oximeter sensor by calibration.

This lack of accurate models can be partially attributed to the fact that even the origin of observed pulsations is far from clear [4]. There are multiple contradictory theories, from traditional volumetric (absorption-based) to scattering-based, which assume rouleau formation and disintegration during the cardiac cycle [11].

This uncertainty is particularly obvious for remote PPG (rPPG), which has a very shallow sampling depth. In particular, while contact pulse oximetry uses spatially resolved measurements, which sample tissues at different depths, the rPPG is an imaging geometry that samples primarily the epidermis and the papillary dermis.

This paper aims to lay the foundations for the theoretical framework, which explains the possibility of extracting tissue blood oxygenation in rPPG geometry. The primary contribution of the article is the model, which directly links the peripheral oxygen saturation with the experimentally measurable value (ratio-of-ratios) in rPPG geometry. In addition, we explored the possibility of assessing the origin of the pulsating volume from experimental data.

## 2. Materials and Methods

Photoplethysmographic techniques assess changes in the blood volume caused by pulse propagation. We will refer to the variable blood volume in microcirculation during the cardiac cycle as a pulsatile volume. More specifically, we will denote an excess blood volume over diastolic volume as a pulsatile volume *V*. Generally, *V* can be expressed as volume per vessel (μm^3^). In this case, the distribution of the pulsatile volumes can be characterized by surface density *ρ* (L/mm^2^). Alternatively, *V* can be expressed as volume per unit of skin surface area, (μm^3^/mm^2^). Moreover, *V* depends on time *V*(*t*). However, we will skip the time dependence for compactness.

### 2.1. Analytical Model

Let us consider the following volumetric model of blood pulsations in rPPG geometry: A single pulsatile volume, *V*, is located at a certain depth, *Z*. This pulsatile volume impacts light propagation. To account for this impact, we can consider a tissue with diastolic blood distribution as a base-case scenario approximated by a homogenous blood distribution. In addition to that, pulsatile blood volumes are present. As these volumes are small, we can consider them perturbations and follow the perturbation approach developed by Saiko et al. [12]. This approach can be summarized as the following: Firstly, we find the light distribution for the homogeneous semi-infinite space. Then, we consider a light-absorbing heterogeneity with excessive absorption coefficient *δμ_a_* and volume *V*, buried at some depth *Z* as a perturbation. We represent this heterogeneity as a negative point source and look for the light distribution caused by this negative light source. The overall light distribution will be the sum of homogeneous and point-source-induced contributions.

In particular, if the inhomogeneity is located at (0,0,*Z*), then the fluence rate at any point on the surface of the tissue surface (here, we assume cylindrical coordinates) in the presence of mismatched boundary can be found as follows [12]:

(3) $$\varphi_{s}(r)=\frac{-3\delta \mu_{a}\mu_{tr}\varphi (Z)V}{4\pi }\left[\frac{\exp(-\mu_{eff}{\left(Z^{2}+r^{2}\right)}^{1/2})}{{\left(Z^{2}+r^{2}\right)}^{1/2}}-\frac{\exp(-\mu_{eff}{\left({\left(2h+Z\right)}^{2}+r^{2}\right)}^{1/2})}{{\left({\left(2h+Z\right)}^{2}+r^{2}\right)}^{1/2}}\right].$$

Here, *φ*(*Z*) is the fluence rate for the homogeneous media (unperturbed solution), *r* is the distance on the surface of the tissue from the projection of the defect to the surface ($r=\sqrt{x^{2}+y^{2}}$), $\mu_{eff}=\sqrt{\mu_{a}/\delta }$, $\delta =1/3\mu_{tr}$,$\mu_{tr}=\mu_{a}+\mu_{s}(1-g)$, and $h=2\delta \frac{1+r_{10}}{1-r_{10}}$, where *r*_10_ is the coefficient of reflection of diffuse light on the border of tissue and air.

Unlike [12], which focused on detecting a single or double inhomogeneity, we are interested in the combined effect of multiple pulsatile volumes distributed homogeneously in the dermis. Thus, we can change perspective, select an observation point (which, for convenience, can be at the beginning of coordinates), and sum contributions from all heterogeneities in the tissue to the fluency rate at this point.

(4) $$\varphi_{s}=2\pi \rho \int_{0}^{\infty }\varphi_{s}\left(r\right)rdr$$

Here, *ρ* is the 2D inhomogeneity density in the plane.

After fairly straightforward integration by applying *u* = *r*^2^, *a* + *u* -> *u*, and $v=\sqrt{u}$ substitutions, we will obtain

(5) $$\varphi_{s}=\frac{-3\delta \mu_{a}\mu_{tr}\varphi (Z)\rho V}{2\mu_{eff}}\exp(-\mu_{eff}Z)\left[1-\exp(-2\mu_{eff}h)\right].$$

As the pulsatile volume *V* changes in time, Equation (5) is directly associated with an *AC* component of the rPPG signal. As such, we can try to estimate the *AC*/*DC* ratio, which is routinely used in PPG measurements. We notice that the *DC* component will have a homogeneous fluence rate at the surface *φ*(0). Thus, the *AC*/*DC* ratio can be found as

(6) ACDC=3δμ×μtr2μeffφ(Z)φ(0)ρVmaxexp(−μeffZ)1−exp(−2μeffh).

Here, *φ*(0) and *φ*(*Z*) are homogeneous fluence rates at the surface and the pulsatile volume’s depth, accordingly. A light-absorbing defect is characterized by excessive absorption coefficient *δμ*, maximum pulsatile volume, *V_max_*, depth *Z*, and surface density *ρ*.

#### Wide Beam Diffuse Illumination

The homogeneous fluence rate within the tissue depends on the illumination. For example, the homogeneous fluence rate can be found using a diffuse approximation for collimated and diffuse illumination.

For a practical case of wide beam diffuse illumination φ(Z)φ(0)=exp(−μeffZ) (see, for example, [13]). Thus, for certain practical applications, Equation (6) can be simplified into

(7) ACDC=3δμ×μtr2μeffρVmaxexp(−2μeffZ)1−exp(−2μeffh).

Consequently, the “ratio-of-ratios”, which is used for blood oxygen saturation calculations, can be written as

(8) R=AC1DC1AC2DC2=δμ1μtr,1δμ2μtr,2μeff,2μeff,1(1−exp(−2μeff,1h1))(1−exp(−2μeff,2h2))exp(−2(μeff,1−μeff,2)Z).

Subindexes 1 and 2 refer to different wavelengths.

In linear approximation on *μ_eff_h*, we will have a much simpler expression.

(9) R=AC1DC1AC2DC2=δμ1δμ2exp(−2(μeff,1−μeff,2)Z)

Note that *R* in Equations (8) and (9) explicitly (and quite strongly) depends on *Z*. Thus, Equations (8) and (9) can potentially be used to extract the depth of pulsations from experimental data.

### 2.2. Simulations

To verify the model, we performed simulations using Equation (8). The following parameters were used.

#### 2.2.1. Absorption

In the absence of melanin, the absorption of the bloodless tissue can be modeled as the background absorption of human flesh [14]: $\mu_{a}=\mu_{a,fl}$ where $\mu_{a,fl}=7.84\times 10^{7}\lambda^{-3.255}$ (mm^−1^). Here, the wavelength λ is measured in (nm).

In the presence of blood (dermis), the absorption of the dermis can be modeled as a combination of background-, oxyhemoglobin-, and deoxyhemoglobin-related absorption.

(10) $$\mu_{a}=(1-c)\mu_{a,fl}+c(SO2\times \mu_{a,HbO2}+(1-SO2)\times \mu_{a,RHb})$$

Here, *c* is the blood volume fraction in the dermis, *SO*2 is the tissue oxygen blood saturation, and HbO2 and RHb refer to oxyhemoglobin and deoxyhemoglobin, respectively. The absorption coefficients for oxyhemoglobin- and deoxyhemoglobin are wellknown [15]. Blood typically occupies around 0.4% of the physical volume of the papillary dermis [16].

However, the tissue oxygen blood saturation will be average between arterial and venous compartments. Thus, Equation (10) can be rewritten taking into account relative volumes of the arterial and venous compartment: ν*_a_* and ν*_v_* (ν*_a_* + ν*_v_* = 1)


(11) $$\begin{array}{l} \mu_{a}=(1-c)\mu_{a,fl}+c\nu_{a}(S_{a}O2\times \mu_{a,HbO2}+(1-S_{a}O2)\times \mu_{a,RHb})+\\ c\nu_{v}(S_{v}O2\times \mu_{a,HbO2}+(1-S_{v}O2)\times \mu_{a,RHb}) \end{array}$$

*S_a_O*2 and *S_v_O*2 are the arterial and venous compartment oxygenations, respectively. Assuming no accumulation of blood into the papillary plexus, the relative blood volume will be approximately equal between compartments, ν*_a_* = ν*_v_* = 1/2. Thus, we can write

(12) $$\mu_{a}=(1-c)\mu_{a,fl}+c\mu_{a,bl}$$

where

(13) $$\mu_{a,bl}=(\frac{S_{a}O2+S_{v}O2}{2})\times \mu_{a,HbO2}+(1-\frac{S_{a}O2+S_{v}O2}{2})\times \mu_{a,RHb}.$$

#### 2.2.2. Scattering

The reduced scattering coefficient for the dermis and epidermis also follows the power law [17] $\mu \prime_{s}\propto \lambda^{-k}$, with *k* = 1.3. With this power law, we can set a reference value at a particular wavelength and simulate its dependence on the wavelength. In particular, we can assign values at 633 nm [18] for the living epidermis (*μ_s_*’ = 9 mm^−1^) and reticular dermis (*μ_s_*’ =5 mm^−1^), which represent the bulk of the tissue in healthy epidermis and dermis, respectively. Assuming normal skin, where the stratum corneum is thin, we can ignore the presence of the stratum corneum. Thus, we can write $\mu \prime_{s}=2.2\times 10^{4}\lambda^{-1.3}$.

#### 2.2.3. Other Parameters

The refractive index of the tissue depends on the wavelength. However, this dependence is small; we will ignore it in our calculations. As such, the refractive index was set to *n* = 1.42. The depth of the pulsating volume was set in the papillary dermis (*Z* = 0.5 mm).

The model depends on two variables: *S_a_O*2 and *S_v_O*2. To simplify interpretation, the assumption was made that the oxygen’s constant fraction (0.3) is extracted during gas exchange in capillaries irrespective of initial oxygenation. Thus,

(14) $$S_{v}O2=S_{a}O2-0.3.$$

As a result, all obtained results depend on a single variable, *S_a_O*2.

#### 2.2.4. Test Scenarios

While it is typically assumed that the pulsatile volume is located on the arterial side, there is no definitive justification. While the arterial side is characterized by the larger changes in the blood pressure, it is also characterized by much smaller compliance than the venous compartment. Thus, it is hypothetically possible that the pulsatile volume is located in the venous compartment.

Thus, to understand the origin of the photoplethysmographic signal, it is necessary to estimate the depth of the pulsations and the compartment where these pulsations occur: arterial or venous. Thus, we have simulated two scenarios: (a) the pulsatile volume is located on the arterial side, and (b) the pulsatile volume is located on the venous side.

In the first scenario, the pulsatile volume is characterized by the arterial oxygenation *S_a_O*2. Thus,

(15) $$\delta \mu_{a}=S_{a}O2\times \mu_{a,HbO2}+(1-S_{a}O2)\times \mu_{a,RHb}-\mu_{a}.$$

In the second scenario, the pulsatile volume is characterized by the venous oxygenation *S_v_O*2. Thus,

(16) $$\delta \mu_{v}=S_{v}O2\times \mu_{a,HbO2}+(1-S_{v}O2)\times \mu_{a,RHb}-\mu_{a}.$$

## 3. Results

**Figure 1:**
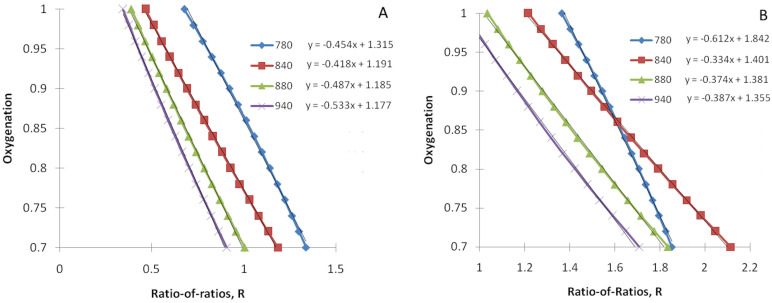
Tissue arterial blood oxygenation as a function of theratio-of ratios *R* for the constant red light wavelength (λ_1_ = 660 nm) and several infrared light wavelengths (780, 840, 880, and 940 nm). (**A**) The pulsatile volume is on the arterial side, and (**B**) the pulsatile volume is on the venous side. The fitting functions are displayed for each R/IR pair. Both oxygenation (SpO2) and the ratio-of-ratios (R) are dimensionless. Oxygenation (SpO2) ranges from 0 to 1 and can be converted to typical clinical presentation (%) by multiplying by 100.

We have simulated the ratio-of-ratios R as a function of tissue arterial blood oxygenation (Equation (13) with Equation (14) as a constraint) for several commonly used infrared light wavelengths (780, 840, 880, and 940 nm), assuming a constant red light wavelength (λ_1_ = 660 nm). As described in Section 2.2.4, we have simulated two scenarios: (a) the pulsatile volume is on the arterial side, and (b) the pulsatile volume is on the venous side. They are depicted in Figure 1A,B, respectively. Note that variables in Figure 1 are transposed in line with the common way of displaying these data (SpO2 as a “ratio-of-ratios” function).

We also performed a linear fit of SpO2 as a function of the “ratio-of-ratios”. The results are also displayed in Figure 1.

As we found that the linear function fits simulated data well (*R*^2^ = 0.98–0.99 for all considered R/IR pairs), we have obtained explicit expressions for the parameters of this linear model, *C*_1_ and *C*_2_. Their derivation is provided in Appendix A.

## 4. Discussion

We derived an explicit analytical relationship connecting blood oxygenation with rPPG geometry’s ratio-of-ratios. We also fitted the obtained function with a linear function. Our first finding was that a linear function in the considered oxygenation range (S_a_O2 = 70–100%) provides an extremely good fit (*R*^2^ = 0.98–0.99) for all considered R/IR pairs.

We can compare our results with experimental data from the literature. In particular, Verkruysse et al. [4] calibrated rPPG oximetry with illumination at 660 and 840 nm by video recordings of the foreheads of 41 healthy adults subjected to normoxic, hypoxic, and low-temperature conditions. They obtained a *SO*2 = 118 − 45.9*R* fitting function. These values are very close to our out-of-the-box prediction for *C*_1_ and *C*_2_ for the 660/840 pair 119.1 and 41.8, respectively (see Figure 1A).

We have analyzed the sensitivity of the *C*_1_ and *C*_2_ to model parameters. The model is insensitive to changes in volume blood fraction c and refractive index n. However, it is very sensitive to the depth of the pulsatile volume *Z*. In particular, for *Z* = 0.6 mm, for *C*_1_ and *C*_2_, we obtained 119.2 and 45.2, respectively, which is very close to values estimated by Verkruysse et al. [4]. Depending on the epidermis thickness, this depth can be in the papillary dermis, subpapillary vascular plexus, or upper portion of the reticular dermis. However, we can estimate more accurately as measurements were taken from the forehead. For example, Jeong et al. [19] found that the epidermal thickness on the forehead is 0.334 ± 0.157 mm. As the thickness of a papillary dermis is approximately 300–400 μm [20], we can conclude that the pulsatile volume is located in the lower part of the papillary dermis or subpapillary vascular plexus.

Another conclusion from this experimental comparison is that pulsatile volume most likely resides on the arterial side. In particular, the venous side’s origin would be characterized by a much smaller coefficient *C*_2_ (see Figure 1B vs. Figure 1A).

Based on these estimations, the pulsatile volume is located in the arterial compartment of the papillary dermis’ lower part or subpapillary vascular plexus. Potentially, it can be an upper portion of ascending arterioles. However, a subpapillary vascular plexus origin is much more likely as it most likely has the largest compliance.

The shortcoming of the proposed model is that it does not account for the impact of the stratum corneum and skin tone. In particular, there is growing evidence that skin tone may impact the accuracy of pulse oximetry measurements [21]. Similarly, the stratum corneum thickness may impact the accuracy of pulse oximetry measurements. In particular, while the thickness of the dermis is quite uniform across all body parts, the epidermis thickness varies between glabrous and non-glabrous skin [22], primarily due to the differences in stratum corneum thickness. Moreover, the stratum corneum thickness can be even higher in corns and calluses. However, we excluded epidermis effects (skin tone and stratum corneum thickness) from consideration as semi-quantitative analysis shows that their impact is nonmaterial. In particular, we can write the ratio-of-ratios *R* as

(17) R=ΔI1I1ΔI2I2=Δln⁡(I1)Δln⁡(I2).

Here, *I*_1_ and *I*_2_ refer to the intensity of the red and infrared light on the sensor, operator Δrefers to changes between systole and diastole. Then, using the Beer–Lambert law for multilayer tissue ($I=I_{0}\exp(-\sum_{i}\mu_{a,i}L_{i}$); here, *L_i_* is the pathlength in the *i*-th layer), we can write

(18) $$R=\frac{\sum_{i}\Delta \mu_{a,1,i}L_{1,i}+\sum_{i}\mu_{a,1,i}{\Delta L}_{1,i}}{\sum_{i}\Delta \mu_{a,2,i}L_{2,i}+\sum_{i}\mu_{a,2,i}{\Delta L}_{2,i}}$$

Here, *μ_a_*_,1,*i*_, *μ_a_*_,2,*i*_ and Δ*μ_a_*_,1,*i*_, Δ*μ_a_*_,2,*i*_ are absorption coefficients for red and IR wavelengths in the *i*-th layer and changes in absorption coefficient between diastole and systole for red and IR wavelength in the i-th layer, accordingly. Similarly, *L*_1,*i*_, *L*_2,*i*_ and Δ*L*_1,*i*_, Δ*L_2_*_,*i*_ are the mean optical path for red and IR wavelengths in the *i*-th layer and changes in the mean optical path between diastole and systole for red and IR wavelengths in the i-th layer, accordingly. Thus, mean optical paths (MOP) are *MOP*_1_ = *∑L*_1,*i*_ and *MOP*_2_ = *∑L*_2,*i*_.

From Equation (17), we can see two types of contributions. In the first term in the nominator and denominator, one can expect that only tissue layers where Δ*μ_a_*_,1,*i*_ and Δ*μ_a_*_,2,*i*_ (pulsatile volume) are non-zero contribute to *R*. The second term in the nominator and denominator accounts for the change in the mean optical path between diastole and systole.

As the surface layers (stratum corneum and living epidermis, where melanin is synthesized) do not contribute to the first terms and are unlikely to have a significant contribution to the second terms, one would expect that surface layers should not provide a meaningful contribution to Equation (17). Thus, it should not have a noticeable impact on the ratio-of-ratios. *R*. However, it does not hold true if the surface layer significantly impacts the passage of light to the dermis (either strong scattering in thick stratum corneum or strong absorption in the melanin layer). In this case, the light does not sample the pulsatile volume, and pulse oxygenation cannot be estimated altogether. Alternatively, the signal is so weak (and noisy) that accurate oxygenation estimation is impossible. For example, it was estimated that oxygenation cannot be accurately extracted for calluses thicker than 1.5 mm [23]. Another effect of the thickening of the epidermis layer is an increase in the pulsatile volume depth, *Z*, and a corresponding increase in the *C*_2_ coefficient (see Equation (A6) for details). Thus, rPPG oxygenation measurement should be taken from skin areas with similar epidermal thicknesses once calibrated. Considering these corner cases, we can conclude that the epidermis should not impact our analysis for light skin tone and not very thick stratum corneum (e.g., non-glabrous skin). However, in the general case, a thorough analysis is required.

In summary, we can conclude that our simple estimation gave us quite realistic values. However, further analysis of the model’s sensitivity to different model parameters is required. In future work, we also plan to validate our results with Monte Carlo simulations, which are a de facto gold standard in tissue optics.

## Data Availability

The original contributions presented in the study are included in the article.

## Appendix A

We can explicitly obtain model parameters *C*_1_ and *C*_2_ of the linear fit model (see Equation (2)) from the developed model. To do so, we can insert expressions for the excessive absorption (Equation (15)) into Equation (8).

(A1)
$$R=\frac{S_{a}O2\times \mu_{a,HbO2,1}+(1-S_{a}O2)\times \mu_{a,RHb,1}-\mu_{a,1}}{S_{a}O2\times \mu_{a,HbO2,2}+(1-S_{a}O2)\times \mu_{a,RHb,2}-\mu_{a,2}}C$$

Here, $C=\frac{\mu_{tr,1}}{\mu_{tr,2}}\frac{\mu_{eff,2}}{\mu_{eff,1}}\frac{\left(1-\exp(-2\mu_{eff,1}h_{1})\right)}{\left(1-\exp(-2\mu_{eff,2}h_{2})\right)}\exp(-2(\mu_{eff,1}-\mu_{eff,2})Z)$; subindexes 1 and 2, as usual, refer to different wavelengths. Here, based on our findings, we assumed that the pulsatile volume resides on the arterial side.

Wavelengths in pulse oximetry are selected in a certain way. Namely, in the red range, the deoxyhemoglobin absorption on several orders of magnitude is larger than those of oxyhemoglobin (and the tissue). Similarly, in the infrared range, the oxyhemoglobin absorption is much higher than deoxyhemoglobin and tissue. Thus, we can extract the leading factors from the nominator and denominator.

(A2)
$$R=\frac{S_{a}O2\frac{\mu_{a,HbO2,1}}{\mu_{a,RHb,1}}+(1-S_{a}O2)-\frac{\mu_{a,1}}{\mu_{a,RHb,1}}}{S_{a}O2+(1-S_{a}O2)\frac{\mu_{a,RHb,2}}{\mu_{a,HbO2,2}}-\frac{\mu_{a,2}}{\mu_{a,HbO2,2}}}\frac{\mu_{a,RHb,1}}{\mu_{a,HbO2,2}}C$$

For compactness, we use the following notation: $C\prime =\frac{\mu_{a,RHb,1}}{\mu_{a,HbO2,2}}C$. From Equation (A2), we can express *S_a_O*2 explicitly:

(A3)
$$S_{a}O2=\frac{C\prime (1-\frac{\mu_{a,1}}{\mu_{a,RHb,1}})+R(\frac{\mu_{a,2}}{\mu_{a,HbO2,2}}-\frac{\mu_{a,RHb,2}}{\mu_{a,HbO2,2}})}{R(1-\frac{\mu_{a,RHb,2}}{\mu_{a,HbO2,2}})+C\prime (1-\frac{\mu_{a,HbO2,1}}{\mu_{a,RHb,1}})}$$

From this expression, we can obtain a linear approximation for *S_a_O*2.

(A4)
$$S_{a}O2=\frac{1-\frac{\mu_{a,1}}{\mu_{a,RHb,1}}}{1-\frac{\mu_{a,HbO2,1}}{\mu_{a,RHb,1}}}-\frac{1}{C\prime }(-\frac{\frac{\mu_{a,2}}{\mu_{a,HbO2,2}}-\frac{\mu_{a,RHb,2}}{\mu_{a,HbO2,2}}}{1-\frac{\mu_{a,HbO2,1}}{\mu_{a,RHb,1}}}+\frac{\left(1-\frac{\mu_{a,1}}{\mu_{a,RHb,1}})(1-\frac{\mu_{a,RHb,2}}{\mu_{a,HbO2,2}}\right)}{{\left(1-\frac{\mu_{a,HbO2,1}}{\mu_{a,RHb,1}}\right)}^{2}})R$$

Thus, we have explicit expressions for coefficients *C*_1_:

(A5) $$C_{1}=\frac{1-\frac{\mu_{a,1}}{\mu_{a,RHb,1}}}{1-\frac{\mu_{a,HbO2,1}}{\mu_{a,RHb,1}}}$$

and *C*_2_:

(A6)
$$C_{2}=\frac{\mu_{a,HbO2,2}}{\mu_{a,RHb,1}}(-\frac{\frac{\mu_{a,2}}{\mu_{a,HbO2,2}}-\frac{\mu_{a,RHb,2}}{\mu_{a,HbO2,2}}}{1-\frac{\mu_{a,HbO2,1}}{\mu_{a,RHb,1}}}+\frac{\left(1-\frac{\mu_{a,1}}{\mu_{a,RHb,1}})(1-\frac{\mu_{a,RHb,2}}{\mu_{a,HbO2,2}}\right)}{{\left(1-\frac{\mu_{a,HbO2,1}}{\mu_{a,RHb,1}}\right)}^{2}})\exp(2(\mu_{eff,1}-\mu_{eff,2})Z)$$

Note the strong dependence of *C*_2_ on *Z*. This dependence can be used to extract the pulsatile volume depth from experimental data.
